# The coronavirus disease of 2019 pandemic-associated stress among medical students in middle east respiratory syndrome-CoV endemic area: An observational study

**DOI:** 10.1097/MD.0000000000023690

**Published:** 2021-01-22

**Authors:** Mohammed A. Batais, Mohamad-Hani Temsah, Hesham AlGhofili, Nawaf AlRuwayshid, Fahad Alsohime, Turky H. Almigbal, Abdulkarim Al-Rabiaah, Ayman A. Al-Eyadhy, Muhammad Hussain Mujammami, Rabih Halwani, Amr A. Jamal, Ali Mohammed Somily

**Affiliations:** a College of Medicine, King Saud University, PO Box 242069; b Department of Family and Community Medicine; c Pediatric Department, College of Medicine; d Prince Abdullah Bin Khaled Coeliac Disease Chair, Faculty of Medicine; e Endocrinology and Metabolism Unit, Department of Medicine, College of Medicine, King Saud University, Riyadh, Saudi Arabia; f Sharjah Institute for Medical Research (SIMR), Department of Clinical Sciences, College of Medicine, University of Sharjah, PO. Box 27272, Sharjah, United Arab Emirates; g Evidence-Based Health Care & Knowledge Translation Research Chair, King Saud University; h Department of Pathology and Laboratory Medicine, College of Medicine, King Saud University Medical City, Riyadh, Saudi Arabia.

**Keywords:** anxiety, coronavirus disease of 2019, distance learning, Medical students, middle east respiratory syndrome -CoV

## Abstract

The coronavirus disease of 2019 (COVID-19) pandemic significantly affected different life aspects, including healthcare communities and academic institutes. We aimed to assess the level of stress and risk factors among medical students and interns during the COVID-19 pandemic in the setting of the middle east respiratory syndrome -CoV endemic area.

A questionnaire-based cross-sectional study was conducted on a randomly selected sample of medical students and interns. The questionnaire was anonymously self-administered to indicate perceive hygienic practice change, importance of viral prevention domestic hygiene, perceive adequacy of received information, perceived agreement to facilitators to alleviate covid stress, self-reported stress level, and generalized anxiety disorder score.

A total of 322 returned the questionnaire (69.7% response rate). Participants had good knowledge regarding severe acute respiratory syndrome -CoV2 in multiple aspects, with an average score of 13.8 out of 14. Two-thirds (62.4%) of the students experienced mild anxiety, (23.9%) had moderate anxiety, (6.8%) had clinically high anxiety level, and another (6.8%) had a clinically very high anxiety level. The stress level, as reported by the respondents (on a 1-10 scale), showed a correlation with the Generalized Anxiety Disorder scale. We observed an increased level of social avoidance and hygienic practice facilitated by availability of hand sanitizers. Majority of the students receive information regarding COVID-19 from reliable and official resources

Most students reported mild to moderate levels of anxiety, and was associated with enhancement of their universal precaution measures. The availability of alcohol-based hand sanitizers and the off-campus study were great relievers. The importance of reliable pandemic resources in educating students during pandemics is emphasized. Furthermore, this study indicate the importance of students’ support services to address mental health and students’ wellbeing in the era of pandemics.

## 1 Introduction

After years of the outbreak of severe acute respiratory syndrome (SARS) and middle east respiratory syndrome (MERS), a new outbreak of respiratory illness caused by a novel member in the coronavirus family has been identified and named coronavirus disease 2019 (COVID-19).^[1]^ Back in 2012, the first case of MERS CoV in Saudi Arabia was isolated.^[1]^ Most of the cases of MERS CoV occurred in Saudi Arabia, with a global mortality rate of 34.4% and 37.1% mortality rate reported in Saudi Arabia in 2019, while ongoing reported cases are being reported in the country until recently^[2]^

On 31 December 2019, the first case of COVID-19 was isolated in Wuhan City, Hubei Province of China, as a case of pneumonia of unknown cause^.^^[3]^ Shortly after, the outbreak spread rapidly through China, impacting the entire country in a matter of 30 days and becoming a public health emergency of international concern as declared by the World health organization (WHO).^[4]^ While COVID-19 can impact people of any age, studies show that patients aged 60 years or older have a higher risk of being infected when compared to children who are less likely to develop the infection or tend only to develop mild symptoms or be asymptomatic.^[5]^ Furthermore, severe disease is more likely to occur in older patients (aged 65 years and older) and those with pre-existing health issues such as chronic lung disease or moderate to severe asthma, heart disease with complications, immunocompromised patients and patients with diabetes, renal failure or liver disease especially if not well controlled.^[6]^ As for the clinical presentation, COVID-19 has a broad spectrum of clinical symptomatology varying from asymptomatic or mild symptoms to acute respiratory distress syndrome and respiratory failure, which requires mechanical ventilation.^[7]^ Most of the confirmed cases have fever, cough (mainly dry), and dyspnea.^[7]^ Other symptoms may occur, including sore throat, runny nose, and gastrointestinal symptoms like nausea and diarrhea.^[8]^

The total number of cases globally exceeded 2 million.^[9]^ Up to date, more than 20,000 cases have been confirmed in Saudi Arabia.^[10]^ Psychological stress is known to occur due to infectious disease outbreaks with significant functional impairment and difficulties in returning to normal daily living after recovery from the disease.^[11]^ Stress can manifest as a change in sleep or eating patterns, difficulty in sleeping or concentrating, or fear and worry about one's health or the health of loved ones.^[12]^ COVID-19 is 1 of the most difficult challenges and threats worldwide, and it has been announced by WHO as pandemic in March.^[8]^

The pandemic has significantly affected different aspects of life, including healthcare communities. Numerous public figures, including actors, athletes, and politicians being infected, and social media rumors are some of the factors that may have caused psychological distress to grow. Healthcare workers (HCW) are facing multiple stressors due to the rapid spread of the disease, the fear of one's own health, and the loved ones. Likewise, medical students and interns are expected to have high-stress levels during the outbreak. High levels of stress can affect them psychologically and may adversely affect their educational process, causing a reduction in their ability to concentrate and ignorance of learning activities. Therefore, in the present study, we assessed the level of stress and risk factors contributes to worry levels experienced by medical students and interns during the COVID-2019 pandemic.

## 2 Methods

### 2.1 Study design and subjects’ selection

This is a cross-sectional study that was conducted during the pandemic of COVID-19 in March 2020 over two weeks period. The college of medicine, King Saud University, has 1176 registered students. During the first two years of study are pre clinical, the thirdyear is an introductory year to clinical settings, while fourth and fifth years are clinical years, where students are more engaged with patients care in wards, clinics, operating rooms, and the emergency room. The target respondents were students from both genders. We divided the responsdents into two groups based on the study year for analysis purposes. Students in their first, second, and third-year were considered junior students, and seniors were those in their fourth, fifth, and intern years. To control for duplicated surveys, 1 leader medical student distributed the survey questionnaire in each academic year. The study was approved by the Institutional Review Board (IRB) at College of Medicine, King Saud University and an informed consent was obtained from participants for the anonymous use of data.

### 2.2 Data collection and processing

The questionnaire was obtained from an earlier published study after the author's permission, then was reviewed by two experts in the field to ensure the validity of the content for currant study.^[13]^ It includes 47 questions categorized under 5 parts. The first part included demographic data like; age, sex, study year, and influenza vaccination status. The second part was conducted to assess the level of changes in personal and social hygiene behaviors with 5 responses (do not know, did not change, changed a little, moderately, or very much). A further question in this section also assessed observed self-change in hand washing, buying sanitizers, avoiding contact with people who have flu symptoms, and avoiding public gathering. The participants were also asked to rate their agreement with several domestic hygiene practices in light of the viral pandemic using a Likert-like scale graded with 5 responses (strongly disagree, disagree, neutral, agree, and strongly agree).

The third part included basic knowledge questions to assess the level of understanding of COVID-19 and its implications. Answers were created to reflect the current literature about COVID-19, its mode of transmission, symptoms, signs, and management. Students were also asked to rate their feelings about access to sufficient information about COVID-19 during the pandemic in terms of symptoms, signs, mode of transmission, prevention, and prognosis. In out of 5 scales, where 5 is “very sufficient.” In addition, we used a 5-points Likert scale to rate their agreement level to multiple statements concerning the current public fear and methods for helping to alleviate the stress induced by the current situation.

In the fourth part, we asked participants about their usual sources of information about COVID-19: WHO reports, Ministry of Health (MOH) official statements and press releases from MOH, MOH website, social media, or college announcements. In the fifth part, we used a 5-responses Likert scale (not worried at all, little worried, somewhat worried, very worried, and extremely worried) to evaluate the participants’ worry concerning public fear during this pandemic. Furthermore, in an out-of-ten scale (10 is the most severe), we asked participants to rate their concern about contracting COVID-19 or transmitting it from the workplace to a family member using a 5-points-Likert scale (5 denoting very worried). This part also included questions about feelings and anxiety about the current COVID-19 pandemic in comparison to the previous MERS-CoV outbreak. Lastly, we conducted the General Anxiety Disorder 7-item (GAD-7) scale. A score of 10 and more is diagnostic of GAD with a sensitivity of 89% and a specificity of 82%.^[14]^

After obtaining the participant's informed consent, an anonymous, self-administered online survey was sent to medical students (years 1–5) and interns via email. Data was collected in a secured Excel TM sheet initially. The confidentiality of the participants was ensured and maintained throughout the study.

### 2.3 Sample size

The sample size was calculated based on previous study findings, which indicate that 77% of medical students reported minimal anxiety toward MERS-COV,^[15]^ with a confidence level of 95% and a margin of error of 5%. By using a standard sample size equation, and assuming balanced responses between groups, the minimum required sample size was estimated to be 377. To overcome the expected incomplete responses, and attrtion rate of 20% to 25%, 462 students were invited to participate in the study.

### 2.4 Statistical analysis

Means and standard deviations were used to describe the continuously measured variables, while frequencies and percentages were used for categorically measured variables. Pearson's correlation test was used to assess the correlation between continuous variables. The Factor analysis dimension reduction technique (Principal components analysis) was used to reduce the various indicators of stress and anxiety from viral infection outbreaks into 1 index, which we named as (stress from viral infection outbreaks factor). The SPSS IBM V21 analysis program was used for the data analysis. The compute command in the analysis program was used to compute the sum score for each of the analyzed concepts by adding up the responses to the indicators comprising these concepts. A Paired samples t-test was used to assess the statistical significance of the mean difference in various facets of stress and anxiety from the current viral COVID-19 pandemic among the students.

Multivariate linear regression analysis was used to assess the combined and individual correlations between the medical student's demographics, academic level, and perceptions of the COVID-19 (independent variables) with their perceived stress from viral outbreaks (dependent variable).

Cronbach test of reliability was used to assess the reliability of the Likert-like scale variables. The alpha significance level was considered at *P* < .05 level for all the statistical tests. The Cronbach alpha test of reliability suggested that the 7 items measuring the students perceived hygienic practices were reliable, Cronbach alpha = 0.872. Likewise, the 4 items that assessed the medical students’ aspects of demoestic hygiene awareness had a Cronbach alpha = 0.74. Also, the 5-item scale measuring the student overall adequacy of information were reliable too, alpha = 0.78. Furthermore, the 4-item scale that measured the alleviating factors that can help reducing stress in current situation of COVID-19, was reliable, Cronbachs alpha = 0.71. The GAD-7 was reliable as well, Cronbach alpha = 0.95.

## 3 Results

### 3.1 Demographic characteristics of participants

A total of 322/462 (69.7%) surveys were completed. Almost half of the respondents were females (53.1%). The mean age for the sample was 21.92 ± 1.81 years. When asked about annual influenza vaccination practice, most of the respondents (77%) ignore this practice and do not get vaccinated. The demographic characteristics and Influenza Vaccination practice of respondents are summarized in Table 1.

**Table 1:** Demographic characteristics and influenza vaccination practice of respondents.

	Frequency	Percentage
Categorical variables		
Sex		
Female	171	53.1
Male	151	46.9
Year of study		
Medical student year 1	46	14.3
Medical student year 2	66	20.5
Medical student year 3	45	14
Medical student year 4	49	15.2
Medical student year 5	83	25.8
Intern	33	10.2
Influenza Vaccination practice		
Yes, I do that every year	50	15.5
Yes, only this year	24	7.5
No	248	77
Continuous variable		
	Mean	SD
Age (yr)	21.92	1.81

### 3.2 Participants perceived indicators of hygienic practice, awareness of domestic health, adequacy of information, public fear as well as factors that can lower stress from COVID-19

Regarding the hygienic practice changes, the top 3 hygienic practice changes reported by the participants were increased avoidance of social visits (mean 3.29 [SD 1.44]), purchase of more sanitizers, (mean 3.2 [SD 1.46]) and avoidance of handshaking (mean 3.18 [SD 1.39]). The mean score of all the 7 indicators was 21.34 ± 6.61 out of 35. (Table 2)

**Table 2:** Descriptive analysis of the medical students’ perceptions of hygienic practices, awareness and adequacy of information in light of COVID19.

	Mean (SD)	Rank
PERCEIVE HYGIENIC PRACTICE CHANGE		
Compliance with hand hygiene at the hospital	2.52 (1.1)	6
Compliance with Universal Precautions (examples: masks and gloves) when in the hospital	3.10 (1.37)	4
Purchasing habits of sanitary items/obtaining hand sanitizer (example: pocket Alcohol Gel)	3.20 (1.46)	2
Avoidance of contact with people having flu symptoms	2.34 (1.21)	7
Avoidance of Social visits (example: visiting friends)	3.29 (1.44)	1
Avoidance of Handshaking habits	3.18 (1.39)	3
Avoidance of the use of public facilities (example: toilets)	3.06 (1.46)	5
IMPORTANCE OF VIRAL PREVENTION DOMESTIC HYGIENE		
It is important to wear face masks in crowded places during influenza or respiratory viral infections season	3.42 (1.2)	4
It is important to maintain good indoor ventilation during flu season to prevent disease spread	3.92 (0.99)	3
It is important to avoid going to crowded places during flu season	4.01 (1.07)	2
Hand hygiene is very important to protect from Corona infection	4.61 (0.92)	1
PERCEIVE ADEQUACY OF RECEIVED INFORMATION ON COVID19 on a scale of 1–5 measuring adequacy		
Symptoms	3.95 (0.98)	3
Prognosis	3.58 (1.08)	4
Treatment	2.9 (1.28)	5
Transmission routes	4.16 (0.91)	2
Prevention	4.21 (0.92)	1
PERCEIVED AGREEMENT TO FACILITATORS TO ALLEVIATE COVID STRESS		
Off-campus studying during outbreaks (online or remote studying)	3.82 (1.12)	2
Educational material from the college/hospital about the COVID-19 prevention	3.55 (1.12)	4
Availability of hand-sanitizers (alcohol gel dispensers) throughout the college/hospital	4.17 (0.92)	1
Presence of hotline support for the students about the outbreak	3.58 (1.20)	3

In regards to the awareness of viral prevention measures, the results yielded by Likert-scale suggested that hand hygiene as a preventive measure to SARS-CoV2 was the most highly rated prevention measure (mean 4.61 ± 0.92 out of 5) followed by the avoidance of crowded places (mean 4.01 ± 1.07 out of 5). Then, in order, the maintenance of good indoor ventilation (mean 3.92 ± 0.99 out of 5) and the importance of wearing face masks in crowded places (mean 3.42 ± 1.2 out of 5). The mean score of the 4 indicators was 15.96 ± 3.14 out of 20. (Table 2)

As for the information received and its adequacy, the participants indicated that the most adequate information was obtained for prevention aspects (mean 4.21 ± 0.92 out of 5 points). This was followed by the modes of transmission of SARS-CoV2 (mean 4.16 ± 0.91 out of 5 points) and the symptoms of COVID-19 (mean 3.95 ± 0.98 out of 5 points). COVID-19 treatment information had the lowest rate (mean 2.90 ± 1.28 out of 5 points). (Table 2)

In addition, most of the participants agreed on the fact that the COVID-19 pandemic resulted in more public fear than the MERS-CoV outbreak and received a rating of (4.39 ± 0.91 out of 5 points). Participants agreed that public fear had increased the awareness of COVID-19 (4.1 ± 0.83 out of 5 points). Less agreement was found on the justification of the public fear (3.15 ± 1.17 out of 5 points). (Table 2)

When asked about the facilitators to alleviate stress due to the COVID19 pandemic, the highest-ranked statement was given to the availability of hand-sanitizers (alcohol gel dispensers) throughout the college/hospital facilities (Mean 4.17 ± 0.92 out of 5 points). This was followed by the off-campus studying during outbreaks, online or remote studying (Mean 3.82 ± 1.12 out of 5 points). Availability of a hotline for support of students during the outbreak and receiving educational material from the college/hospital about COVID-19 prevention were ranked as the least facilitators least likely to alleviate stress due to COVID19 pandemic (Mean 3.58 ± 1.20 out of 5 points) (3.55 ± 1.12 out of 5 points). (Table 2)

### 3.3 Participants’ knowledge of COVID-19 and sources of information

In general, most of the participants had good knowledge regarding SARS-CoV2 in terms of mode of transmission, the disease it causes and the available management options with the average score for all participants being 13.8 out of 14. (Table 3)

**Table 3:** Descriptive statistics of perceptions of COVID-19 among participants.

	Mean (SD)	Median
Knowledge score on COVID19, (maximum possible score 0–14 points)	13.81 (1.13)	14.00
Perceived Hygienic Practices change during COVID19, (7–35 maximum possible points)	21.34 (6.61)	22.00
Perceived Domestic Hygiene awareness COVID19, (4–20 maximum possible points)	15.96 (3.14)	16.00
Perceived adequacy of information on COVID19, (5–25 maximum possible points)	18.80 (3.80)	19.00
Perceived facilitations during COVID19, (4–20 maximum possible points)	15.11 (3.21)	15.00
Over the past 4 weeks, how worried were you from contacting COVID19 yourself 1–5 score	2.60 (1.10)	3.00
Over the past 4 weeks, how worried were you from transmitting corona to your family 1–5 score	3.10 (1.3)	3.00
Generalized Anxiety Score (GAD) 0–23 score	4.92 (5.2)	3
Generalized Anxiety Disorder score classification, n (%)		
Mild: 0–5 points		201 (62.4)
Moderate > 5–10 points		72 (23.9)
High > 10–15 points		22 (6.8)
Very high > 15 points		22 (6.8)

**Figure 1:**
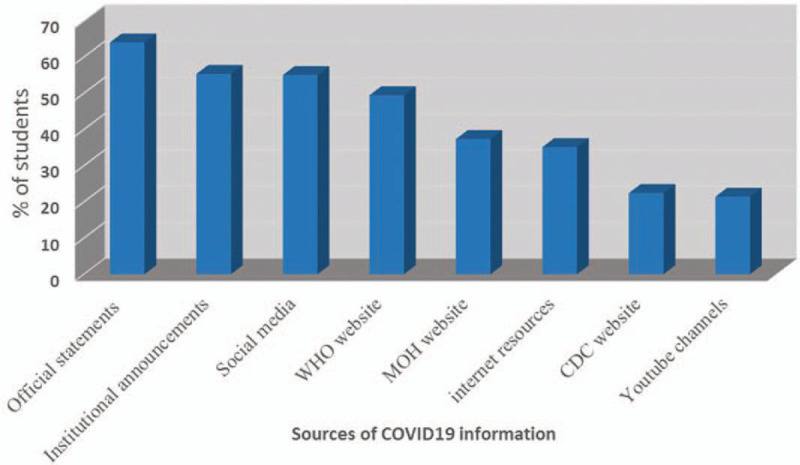
Information accessed resources.

Figure 1 shows the percentages of different information resources among participants. Official statements and press releases by the MOH were ranked as the usual source of information by 64% of the participants. This was followed by college and hospital announcements (55%), and the WHO websites (49%). Internet sources like; blogs, forums, and YouTube channels were indicated as the source used by the fewest participants for information about COVID-19.

### 3.4 The anxiety level of the participants and associated factors

Furthermore, when asking the participants about their stress regarding contracting COVID-19 while training at the hospital, there was a mean score of 2.60 ± 1.10, and their mean stress level of transmitting COVID19 to their families and others was higher with a mean score of 3.10 ± 1.30. In term of GAD score, (62.4%) of the participants had mild anxiety, (23.9%) of them had moderate anxiety, while (12.6%) had clinically either high or very high anxiety levels. (Table 3)

The Pearson's correlation test showed a correlation of the participants stress with their perceived hygienic practices changes during the COVID-19 (*r* = 0.515, *P* < .01), their domestic hygiene practices, (*r* = 0.201, *P* < .01), their required facilitations score, (*r* = 0.31, *P* < .01) and with their GAD7 score, (*r* = 0.48, *P* < .01) (Table 4). All of these correlations were positive, indicating that participants with more behavioral change had higher perceived stress.

**Table 4:** Correlation between stress level and continuous predictors included in the model.

	Stress from Viral outbreaks FACTOR	Knowledge COVID	Hygienic practices change	Domestic hygiene	Sufficiency of information	Perceived facilitations	GAD7
Knowledge score on COVID19	.067						
Hygienic Practices change during COVID19	.515^†^	.058					
Domestic Hygiene Importance awareness COVID19	.201^†^	-.012	.168^†^				
Perceived adequacy of information on COVID19	-.047	-.036	-.051	.247^†^			
Perceived facilitations during COVID19	.312^†^	.013	.295^†^	.109	.084		
Square Root (GAD7)-Generalized anxiety disorder score	.479^†^	.019	.264^†^	.088	-.117^∗^	0	
Number of accessed sources of information	0.031	.010	0.03	.100	.11^†^	.110	.106

The linear regression analysis revealed a significant association between the perceived stress and several characteristics including the male gender (Beta = 1.014, *P* = .003), the academic year of the students (Beta = 0.286, *P* = .006), the GAD7 score (Beta = 1.183, *P* < .001), and the perceived hygienic practice changes (Beta = 1.55, *P* < .001). (Table 5)

**Table 5:** Linear regression model predicting stress level of students as measured on 1–10 points.

	Beta	Std. Error	Standardized Beta	*t*-value	*P*-value
(Constant)	40.177	2.384		16.854	<.001
Sex = Male	1.014	.333	.132	3.048	.003
Year of study	.286	.104	.122	2.744	.006
Takes annual vaccine = Yes	.448	.388	.049	1.154	.249
SQRT-GAD7	1.183	.136	.397	8.689	<0.001
Knowledge score on COVID19.	0.064	.142	.019	0.452	.652
Hygienic Practices change during COVID19	1.550	.187	.382	8.307	<0.001
Perceived Domestic Hygiene awareness COVID19	.580	.216	.119	2.686	.008
Perceived adequacy of information on COVID19	-.215	.222	-.043	-0.969	.334
Perceived required facilitations during COVID19	0.761	0.215	0.158	3.541	<0.001

## 4 Discussion

During the current COVID-19 pandemic, there is an enormous need to understand the perception of frontline workers who are confronting the disease. Frequently, medical students are not considered when discussing frontline workers, although they are a particularly vulnerable population. Understanding the perception of COVID-19 among these students, how the disease has impacted both their outlook and their behaviors, and methods for intervening to protect and inform them is therefore very important. Our study findings provide vital new evidence about how the COVID-19 is impacting medical students who are at the frontline of this pandemic. The results from this survey indicate that while knowledge of COVID-19 among these students is very high, there is still substantial concern about the disease. For example, students changed their hygienic practices due to the disease and were particularly worried about passing on the illness to their family members, rather than contracting the disease themselves. The same fear was reported during the SARS outbreak in Canada, where the staff was adversely affected by transmitting the disease to their families.^[16]^ However, as opposed to MERS-CoV, HCWs were less worried about transmitting the disease to their families, which may be due to the higher transmissibility SARS-CoV 2.^[17]^

**Figure 2:**
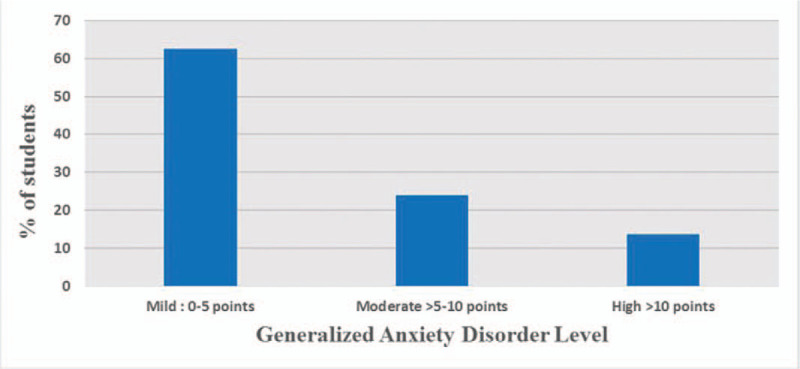
GAD-7 anxiety level category.

Generally speaking, the healthcare workers, including physicians, nurses, medical students or interns, and others who work in hospitals are at high risk of contracting the infection, and this high risk may be another cause of the increasing levels of their psychological distress. In Italy, around 20% of HCWs who responded to the pandemic contracted the disease, and some have lost their lives. Whereas in China, around 3000 cases have been confirmed among HCWs.^[18]^ As the pandemic spreads rapidly and affects large numbers daily, a general state of anxiety is commonly experienced as it is with other cases of emergent diseases. GAD-7 is a 7-item scale with high sensitivity in screening for GAD; individuals who score more than 10 in this scale were found to have GAD. We have used this scale to remove the effect of any underlying pathological anxiety in our sample. Further, the stress level reported by our study subjects correlated significantly with their GAD score (Fig. 2).

With this understanding that COVID-19 is a concern among these students and impacting their behaviors, it is important to understand what factors, in particular, may be associated with stress among these students and how this stress may be changing behaviors. The findings from the survey indicated that students reporting more stress were also more likely to report more changes in these hygienic practices. These findings may be positive in the sense that they indicate that students are channeling their stress into changing health behaviors. At the same time, these findings are concerning, because it may indicate that there is a group of students reporting low levels of stress which are not substantially changing their hygienic process, which could hamper efforts to control the spread of the disease. Low level of stress may be due to younger students perceiving themselves as being likely not to be affected by the disease, which has had its most serious impacts among older populations. It may be important for messaging around COVI-19 to emphasize that this infection can make them very sick or transmitted through them to more volunerable family member or person who might develop serious complication or die.

In contrast to previous research where junior students tended to worry more,^[19]^ in our study, years of study were associated with higher stress levels. These findings may be due to a few different factors. Students in later years of study are more likely to be engaged in clinical practice and therefore are more likely actually to be exposed to patients with COVID-19. Additionally, because these students likely started their studies closer to the MERS outbreak, the memory of that outbreak may be in their mind, therefore making them feel more stressed about the present pandemic. Choi et al, stated that the imbact of COVID-19 is particularly more on students who transit to become doctors, since the effect is more on the students clinical electives which may affect there confiedence and preparedeaness.^[20]^

The findings from this study also provide some evidence about the best methods for communicating information about COVID-19 to this population of medical students. In our sample, students were more likely to take the information from the MOH official statements and press, followed by the institutional announcement. These findings are positive because they suggest that the students are getting information from trustworthy and vetted resources. This is opposite to the sources that were used during the MERS-CoV outbreak in the same setting, where the medical students obtained their information from the MOH sources in only 22.8%.^[15,21]^ Generally, Saudi population has good knowledge about multiple aspects in regards to the COVID-19 and they showed better understanding than Chinese population.^[22,23]^ This can be explained by the changes exhibited by the MOH media center during the COVID-19 pandemic, where all the announcements are provided daily and firstly through the official statements. Also, these results reflect the importance of the official statements and college announcements in students teaching about the disease during infection outbreaks. More concerningly, a lot of the students indicated that they get information from social media. This source of information can be an issue because this data may not be vetted and trustworthy. It has been documented that there have been substantial amounts of misinformation of COVID-19 shared over social media. Efforts should be put in place to counter any false narratives about the disease.

Fortunately, students seem to overall be satisfied with the amount of information that they have received about symptoms, prognosis, and prevention of COVID-19. Students reported being less satisfied with having information about the treatment of COVID-19. This finding may not necessarily be reflective of information about treatment not being disseminated, but instead reflective of the fact that there is still not a lot of information available about the best methods for treating COVID-19 and that is consistant with a study done in Turkey where most of students wanted only to know when and how the pandemic will end.^[24]^ Thus, we believe it is important for students to be given scientific predictions.

There is also positive evidence from this survey that students are changing their behaviors in response to the pandemic. In particular, medical students indicate that they are making substantial changes to avoid social visits, purchase sanitary items, and avoid handshaking. This improvement is explained by their knowledge about the importance of hand hygiene and avoidance of social contact when we asked them about the viral prevention measures. Further, this is in line with an earlier published study done in the same institution on HCW during the MERS-CoV outbreak. They reported an overall increase in adherence to universal precautions.^[25]^

The strength of our study lies in the study settings. Hawryluck et al study reported the psychological imbact of quarantine on general population and found that 28.9% of people exhibited posttraumtic stress disorder and 31% had depression.^[26]^ Furthermore, Meo et al found that 23.5% of medical students felt disheartend and depressed and they relied the reason behind the psychological distress to that people are having an unpleasant experience, students have to depart from fellows, friends and family and losing the ability to move about freely.^[27]^ Our study was conducted during the COVID-19 pandemic when the number of cases globally was hundreds of thousands. When national and international public fear reached high levels due to the uncertainties in multiple aspects of this virus. Senior medical students have graduated earlier than their expected graduation dates and were involved in patient care in some countries.^[28]^ In such situations, approximately two-thirds of the students in our sample had mild anxiety levels, and that can be explained by the low perceived risk of COVID-19 and the high level of knowledge. However, our results of stress levels may underestimate the actual level because the government acted proactively and provided off-campus education to all students. So, medical students during the time of study did not have direct patient contact. When comparing the difference in stress level between genders, we found no significant difference between males and females, and this is consistent with earlier study concerned with stress level during the SARS epidemic.^[29]^ Most of the students had described the adequacy of information regarding treatment as low, so this may also explain the level of anxiety, as they have a fear of the unknown. So, we recommend providing up-to-date information to students during the pandemic. This is similar to the

Our study has a number of limitations related to the study design. The use of a convenience sample of a single location means that the findings of this study cannot necessarily be generalized to other Saudi Arabian university students. The sampling strategy may also have resulted in response bias, with medical students who were more interested in the subject of COVID-19 being more likely to participate. Therefore, further research on a larger scale and employing methods that are more diverse is needed in order to address these shortcomings.

## 5 Conclusion

The findings from this study indicate that there have been substantial behavior changes among medical students in Saudi Arabia. This behavior change will hopefully help to protect the students themselves, their patients, and others that they come in contact with. Students seem to be getting a lot of information from official and institutional sources. Efforts should be made to ensure that the latest information is always being shared with these students. Additionally, efforts should be made to combat false information that is shared on social media, as many students indicated that they get information from social media.

## Acknowledgments

The authors are grateful to the Deanship of Scientific Research, King Saud University for funding through Vice Deanship of Scientific Research Chairs.

## Author contributions

**Conceptualization:** Mohammed A. Batais, Mohamad-Hani Temsah, Nawaf AlRuwayshid, Turky H. Almigbal, Abdulkarim Al-Rabiaah, Ayman A. Al-Eyadhy, Muhammad Hussain Mujammami, Rabih Halwani, Ali Mohammed Somily.

**Data curation:** Mohammed A. Batais, Mohamad-Hani Temsah, Turky H. Almigbal, Abdulkarim Al-Rabiaah, Ayman A. Al-Eyadhy, Muhammad Hussain Mujammami, Rabih Halwani.

**Formal analysis:** Mohammed A. Batais, Mohamad-Hani Temsah, Nawaf AlRuwayshid, Muhammad Hussain Mujammami, Rabih Halwani, Amr Jamal.

**Investigation:** Hesham AlGhofili, Nawaf AlRuwayshid, Fahad Alsohime, Turky H. Almigbal, Abdulkarim Al-Rabiaah, Muhammad Hussain Mujammami, Rabih Halwani, Amr Jamal.

**Methodology:** Mohamad-Hani Temsah, Hesham AlGhofili, Nawaf AlRuwayshid, Fahad Alsohime, Turky H. Almigbal, Abdulkarim Al-Rabiaah, Ayman A. Al-Eyadhy, Amr Jamal, ALI MOHAMMED SOMILY.

**Project administration:** Mohammed A. Batais, Ali Mohammed Somily.

**Resources:** Fahad Alsohime.

**Supervision:** Mohammed A. Batais, Ali Mohammed Somily.

**Visualization:** Hesham AlGhofili.

**Writing – original draft:** Mohammed A. Batais, Hesham AlGhofili, Nawaf AlRuwayshid, Fahad Alsohime, Ayman A. Al-Eyadhy, Amr Jamal.

**Writing – review & editing:** Mohammed A. Batais, Mohamad-Hani Temsah, Ali Mohammed Somily.
